# Public health economic modelling in evaluations of salt and/or alcohol policies: a systematic scoping review

**DOI:** 10.1186/s12889-024-21237-7

**Published:** 2025-01-08

**Authors:** Joseph Prince Mensah, Chloe Thomas, Robert Akparibo, Alan Brennan

**Affiliations:** Sheffield Centre for Health and Related Research (SCHARR), University of Sheffield, Sheffield, UK

**Keywords:** Modelling, Salt, Sodium reduction, Alcohol, Scoping review, Non-communicable diseases, Cardiovascular disease

## Abstract

**Background:**

Public health economic modelling is an approach capable of managing the intricacies involved in evaluating interventions without direct observational evidence. It is used to estimate potential long-term health benefits and cost outcomes. The aim of this review was to determine the scope of health economic models in the evaluation of salt and/or alcohol interventions globally, to provide an overview of the literature and the modelling methods and structures used.

**Methods:**

Searches were conducted in Medline, Embase, and EconLit, and complemented with citation searching of key reviews. The searches were conducted between 13/11/2022 and 8/11/2023, with no limits to publication date. We applied a health economic search filter to select model-based economic evaluations of public health policies and interventions related to alcohol consumption, dietary salt intake, or both. Data on the study characteristics, modelling approaches, and the interventions were extracted and synthesised.

**Results:**

The search identified 1,958 articles, 82 of which were included. These included comparative risk assessments (29%), multistate lifetables (27%), Markov cohort (22%), microsimulation (13%), and other (9%) modelling methods. The included studies evaluated alcohol and/or salt interventions in a combined total of 64 countries. Policies from the UK (23%) and Australia (18%) were the most frequently evaluated. A total of 58% of the models evaluated salt policies, 38% evaluated alcohol policies, and only three (4% of included modelling studies) evaluated both alcohol- and salt-related policies.

The range of diseases modelled covered diabetes and cardiovascular disease-related outcomes, cancers, and alcohol-attributable harm. Systolic blood pressure was a key intermediate risk factor in the excessive salt-to-disease modelling pathway for 40 (83%) of the salt modelling studies. The effects of alcohol consumption on adverse health effects were modelled directly using estimates of the relative risk of alcohol-attributable diseases.

**Conclusions:**

This scoping review highlights the substantial utilisation of health economic modelling for estimating the health and economic impact of interventions targeting salt or alcohol consumption. The limited use of combined alcohol and salt policy models presents a pressing need for models that could explore their integrated risk factor pathways for cost-effectiveness comparisons between salt and alcohol policies to inform primary prevention policymaking.

**Supplementary Information:**

The online version contains supplementary material available at 10.1186/s12889-024-21237-7.

## Background

 Non-communicable diseases (NCDs), such as cardiovascular diseases (CVDs) and cancers, are caused, to some extent, by modifiable risk factors such as unhealthy diets, excessive salt intake, harmful use of alcohol, and physical inactivity [1]. The excessive intake of dietary salt accounts for a large increase in hypertension prevalence since it is associated with an increase in systolic blood pressure [2]. The unhealthy consumption of alcohol has also previously been linked to acute and chronic changes in systolic blood pressure, and studies suggest that alcohol consumption can cause hypertension in heavy drinkers [3]. These two diet-related risk factors for hypertension and non-communicable diseases are therefore responsible for significant health and economic burden globally [1]. One way to reduce this burden is by implementing public health policies to control the consumption of these risk factors in the population [1, 4].

Health economic modelling has widely been used to generate scientific evidence on the costs and outcomes of dietary salt-reduction interventions and alcohol policies, aiding the evaluation and planning of dietary and other public health strategies [5]. Recent reviews have focused on health economic models for population-level dietary policies or alcohol policies, offering policymakers an overview of available health economic models to adapt or implement [6–8]. However, some of these reviews have excluded interventions at the primary care level and have not examined models addressing both salt and alcohol together. To our knowledge, no review has comprehensively explored the scope of health economic models evaluating policies targeting excessive dietary salt intake, alcohol consumption, or both. Identifying and understanding the scope of models addressing both salt and alcohol consumption will support the evaluation of interventions for efficient resource allocation and priority setting, while offering insights into common modelling approaches and health outcomes to inform the development of joint models targeting both risk factors.

This study systematically reviews the literature to identify health economic modelling studies evaluating public health interventions or policies targeting the harmful alcohol use and/or excessive intake of dietary salt. The aim is to assess the global scope and volume of these studies, provide an overview of their modelling structures, and analyse the role of intermediate risk factors in the modelling of salt or alcohol consumption to related health outcomes.

## Methods

### Study design

The scoping review was conducted following the reporting standards in the PRISMA extension for scoping reviews (PRISMA-ScR) [9]. A systematic scoping review aims to “map the key concepts underpinning a research area and the main sources and types of evidence available” [10]. For this scoping review, we defined public health economic models as decision analytic models or mathematical tools used in economic evaluations to compare and synthesise evidence on costs and health benefits [11].

### Search strategy

A comprehensive search strategy was developed and used to identify all potentially relevant peer-reviewed studies published in English. The search strategy was tested, refined and subsequently used to conduct systematic searches in the following electronic databases: Medline, EMBASE (via Ovid), and EconLit (via Ovid). The search was first performed on 13/11/2022, with an update search conducted on 8/11/2023 (see Additional file 1). The search covered publications from 01 January 1990 to the updated search date (i.e., 8/11/2023). The search terms utilised four broad categories of terms, combined using Boolean operators: (salt OR alcohol) AND economic evaluation AND modelling. The search terms used were complemented by MeSH terms, and the NHS CRD EED search filter for economic evaluation [12] was applied in the Medline and EMBASE searches.

The search records were imported into the Covidence reviewing tool by Cochrane for screening. Two reviewers (JPM, JO) independently screened all titles and abstracts of the identified studies against the inclusion criteria, and discrepancies were resolved by consensus. The full texts of the remaining studies were subsequently assessed to determine their eligibility for inclusion. Citation searches of five key reviews [6–8, 13, 14], as well as the reference lists of the included studies, were scanned to identify studies that were missed following the database searches.

### Inclusion and exclusion criteria

A study was deemed eligible for inclusion if it was a model-based full health economic evaluation, i.e., a comparative analysis of alternative health interventions/policies in terms of both costs and consequences [15]. We considered population-level and individual lifestyle interventions or policies aimed at reducing the harmful consumption of alcohol, dietary salt, or both unhealthy dietary behaviours in any defined human population globally.

Conversely, studies were excluded based on the following criteria:

1. Economic evaluations were based on empirical trials, i.e., within-trial or alongside randomised controlled trials (RCTs).
2. Evaluations of pharmacological interventions, medical devices or procedures, or diagnostic tests.
3. Epidemiological models that evaluate only the burden of disease with no cost or health economic outcomes.
4. Cost analysis studies that do not examine the consequences or health-related outcomes.
5. Non-English language studies.

### Data extraction and synthesis

A pair of reviewers extracted the relevant data from the included studies using a data extraction template, and disagreements were resolved by consensus. We grouped the included studies into eight categories of model structure by examining their methodological approach based on definitions in Briggs et al. [16]. For each study, we extracted the following data.

- Study description: author, publication year, and country/setting.
- Policy/intervention details: intervention/policy evaluated, and target population.
- Model details: model name, model structure, and cost perspective.
- Study outcomes: health outcomes, cost outcomes, and currency year.

For each study, there was a particular focus on the model methodology and structure. We synthesised the extracted model characteristics to not only provide an overview of the included models but also to describe the modelled underlying risk factor to disease pathway, the effect of interventions on the model pathway to disease, and the approaches used to calculate health and economic outcomes.

## Results

### Search results

**Fig. 1:**
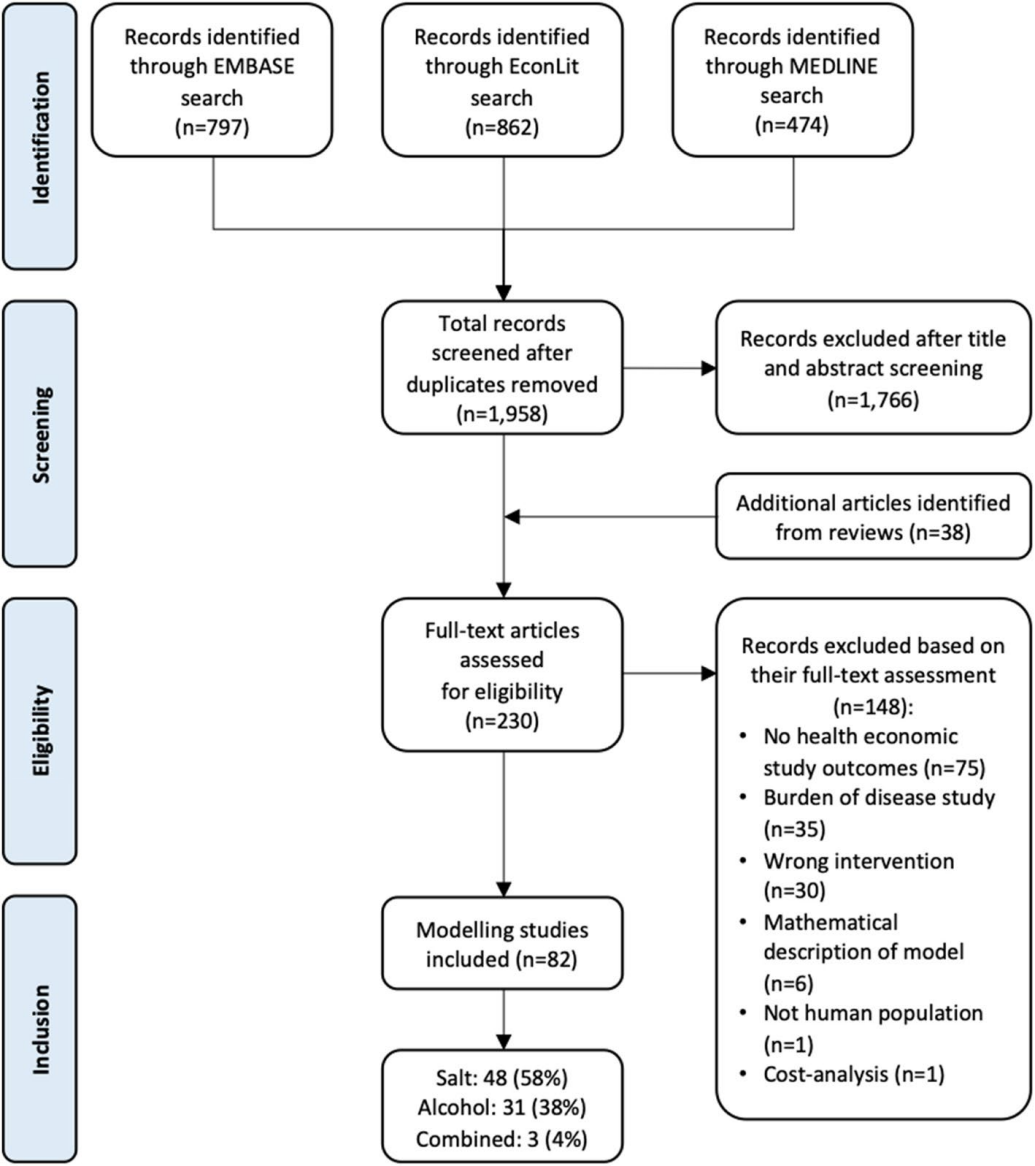
PRISMA-ScR flowchart outlining the inclusion and exclusion of the identified studies

The search yielded a total of 1,958 articles after duplicates were removed. Only 230 full-text articles potentially met the eligibility criteria after title and abstract screening. After full-text assessment of the 230 articles against the inclusion criteria, 148 additional articles were removed, leaving a total of 82 health economic modelling studies which were included in the review. Figure 1 presents a PRISMA flow chart describing the selection process. Table 1 presents a summary of the included health economic models and their study characteristics.

**Table 1:** Summary of the included health economic models

Study Author (Year), Setting	Population	Policy details	Model name	Method/Structure	Health outcomes	Cost outcomes (perspective)
Alcohol policy models						
Brennan et al. (2022), United Kingdom [17]	adults aged 18+	national level minimum unit price policies for alcohol	Sheffield Alcohol Policy Model (Local Authority version 4.0)	comparative risk assessment, lifetable	hospitalisations, crimes and alcohol-attributable deaths and inequalities	NHS costs (healthcare)
Gibbs et al. (2022), South Africa [18]	drinking population aged 15 and older	minimum unit pricing of alcohol	epidemiological policy appraisal model	comparative risk assessment, multistate lifetable	alcohol consumption and expenditures, reduction in cases of catastrophic health expenditures, alcohol-related diseases averted	government healthcare cost savings, household savings linked to workplace absence (healthcare)
Brennan et al. (2021), United Kingdom [19]	population aged 18 years and over	minimum unit pricing of alcohol	Sheffield Alcohol Policy Model (version 4.0)	comparative risk assessment, multistate lifetable	alcohol-attributable deaths, hospitalisations and crime, QALYs	changes in alcohol-attributable NHS costs (healthcare)
Robinson et al. (2020), Australia [20]	population aged 15 years and over	uniform volumetric tax and minimum unit floor price on alcohol	ACE-Obesity Policy Model	multistate lifetable model	Health-adjusted life years	healthcare cost savings (societal)
Jiang et al. (2019), Australia [21]	population aged 16 and over	alcohol pricing policies: tax policy, minimum unit pricing	economic and epidemiological modelling	comparative risk assessment	deaths, DALYs	healthcare costs (healthcare)
Cobiac et al. (2019), New Zealand [22]	general population	increase in alcohol excise taxes	multistate life-table model	multistate lifetable model	QALYs	healthcare costs (healthcare)
Angus et al. (2017), 28 EU member states [23]	general population	national screening and brief interventions programme	meta-modelling approach based on SAPM (Sheffield Alcohol Policy Model)	comparative risk assessment, lifetable	QALYs gained, ICERs	Cost of GP, hospitalisation, net programme cost (healthcare)
Galarraga et al. (2017), Kenya [24]	HIV + persons	task-shifted cognitive-behavioural therapy	Generic	Cohort model	productivity improvements	training and administering task-shifted CBT therapy costs, averted cost from reduced likelihood of HIV transmission (societal)
Zur et al. (2016), Canada [25]	population aged 17 years and older	alcohol screening and brief intervention	microsimulation model of alcohol consumption	microsimulation	life years gained, QALYs gained	direct healthcare costs (healthcare)
Kessler et al. (2015), East Africa [26]	HIV infected persons	cognitive based treatment aimed at reducing hazardous alcohol consumption	computer simulation model	system dynamics model	Welfare-Adjusted Life Years	healthcare costs (healthcare)
Angus et al. (2014), Italy [27]	general population (16+)	Screening and brief interventions	Sheffield Alcohol Policy Model	comparative risk assessment, lifetable	QALY gains	Programme delivery cost and healthcare savings (healthcare)
Brennan et al. (2014), United Kingdom [28]	Adults and young people aged 16 or more, including subgroups of moderate, hazardous, and harmful drinkers	minimum unit pricing	Sheffield Alcohol Policy Model (version 2.5)	comparative risk assessment, lifetable	Changes in mean consumption in terms of units of alcohol, drinkers’ expenditure, and reductions in deaths, illnesses, admissions to hospital, and quality adjusted life years	healthcare costs (healthcare)
Holm et al. (2014), Denmark [29]	adult population aged 16 years or older	30% increased taxation, increased minimum legal drinking age, advertisement bans, limited hours of retail sales, and brief and longer individual interventions	Assessing Cost-Effectiveness in Prevention (ACE-Prevention) model	multistate lifetable model	DALYs averted	inpatient and outpatient costs (healthcare)
Holm et al. (2014), Denmark [30]	adult Danish population aged 16 years and older	changes in alcohol taxation	multistate lifetable model	multistate lifetable model	DALYs	intervention costs (healthcare)
Ditsuwan et al. (2013), Thailand [31]		Random breath testing, selective breath testing, and mass media campaigns	Generalized cost-effectiveness analysis	comparative risk assessment	DALYs averted	intervention costs, treatment cost savings (healthcare)
Purshouse et al. (2013), United Kingdom [32]	population aged 16 years and over	universal alcohol screening and brief intervention programmes in primary care	Sheffield Alcohol Policy Model	comparative risk assessment, lifetable	QALYs	screening costs, programme costs, alcohol-related costs, and NHS costs (healthcare)
Doran et al. (2013), Australia [33]	general population	volumetric tax; equal tax rate to all beverages equivalent to a 10% increase in the current excise applicable to spirits and ready-to-drink products; excise tax rate that increases exponentially by 3% for every 1% increase in alcohol content above 3.2%; and applying a two-tiered volumetric tax	ACE (Assessing the Cost Effectiveness)-Alcohol model	multistate lifetable model	DALYs	healthcare costs averted, taxation revenue (healthcare)
Popova et al. (2012), Canada [34]	general population	privatisation of alcohol sales, compared with government alcohol retailing systems	simulation model	comparative risk assessment	number of deaths, potential years of life lost	direct healthcare costs, indirect costs (productivity losses), direct costs of criminality (societal)
Magnus et al. (2012), Australia [35]	2008 adult cohort aged 15–65 years	realistic target reduction in per capita annual adult alcohol consumption	population simulation model		DALYs	health sector costs, production gains or losses (societal)
Navarro et al. (2011), Australia [36]	Risky drinkers in 10 rural communities in New South Wales, Australia	GP-delivered interventions for alcohol misuse: screening, brief interventions, combination	decision model	decision tree	reduction in alcohol consumption	training and GP costs (healthcare)
Cadilhac et al. (2011), Australia [37]	adult population aged 15 and over	feasible reduction in behavioural risk factors (alcohol)	population simulation model		DALYs, avoidable disease, deaths	health sector costs (societal)
Purshouse et al. (2010), United Kingdom [38]	adults aged 18 years or over	alcohol pricing policies	Sheffield Alcohol Policy Model version 1.1	comparative risk assessment, lifetable	QALYs	healthcare costs (healthcare)
Barbosa et al. (2010), United Kingdom [39]	all males	Motivational enhancement therapy, compared with social behaviour and network therapy	probabilistic lifetime Markov model	Markov model	years of life, QALYs	lifetime treatment costs and disease-related costs (healthcare)
Byrnes et al. (2010), Australia [40]	general population	volumetric alcohol taxation	multistate lifetable model	multistate lifetable model	DALYs averted	cost to government, cost savings, intervention cost (healthcare)
Tariq et al. (2009), the Netherlands [41]	Risky drinkers aged between 20 and 65 who visit the GP yearly	Screening and brief interventions	RIVM Chronic Disease Model	Markov model	QALYs gained	intervention and healthcare costs (healthcare)
Cobiac et al. (2009), Australia [42]	population aged 18 years and over	volumetric taxation, advertising bans, an increase in minimum legal drinking age, licensing controls on operating hours, brief intervention (with and without general practitioner telemarketing and support), drink driving campaigns, random breath testing	multi-state, multiple cohort lifetable approach	multistate lifetable model	DALYs averted	healthcare costs (healthcare)
Van den Berg et al. (2008), the Netherlands and Sweden [43]	entire population	alcohol tax increases	RIVM Chronic Disease Model	Markov model	QALYs gained	healthcare costs (healthcare)
Lai et al. (2007), Estonia [44]	general population	excise tax on alcoholic beverages; reduced access to alcoholic beverage retail outlets; a comprehensive advertising ban (TV, radio and billboards) on alcoholic products; roadside breath-testing for blood alcohol content in motor vehicle driver; and brief interventions involving counselling to at-risk drinkers by a primary care physician	WHO-CHOICE	comparative risk assessment	DALYs	patient and programme costs (societal)
Mortimer et al. (2005), Australia [45]	heavy drinkers aged 19 and over	brief interventions for problem drinking	Time-dependent state-transition model	Markov model	Health-related quality of life (HRQoL) gain	(societal)
Chisholm et al. (2004), 12 epidemiological WHO subregions [46]	Individuals at risk of alcohol use	Interventions to reduce use of alcohol and tobacco use	population model		cost per DALYs averted	(healthcare)
Downs et al. (1995), United States of America [47]	Adolescents aged 15 to 19 years	screening visits for all adolescents and counselling visits for youth identified as high risk	cost-effectiveness model	decision tree	cost-effectiveness	direct and intervention costs (societal)
Salt policy models						
Aminde et al. (2023), Australia [48]	Adult population	Achieving the Australian national sodium reduction targets	proportional multistate lifetable model	multistate lifetable Markov model	Chronic kidney disease incidence, disease deaths, HALYs, life expectancy	Annual health spending for chronic kidney disease (healthcare)
Ikeda et al. (2022), Japan [49]	adults aged 40 to 79 years	achieving global and national salt-reduction targets (8, < 6, and < 5 g/day)	discrete-time Markov cohort macro-simulation model	Markov model	disease incidence	outpatient care and drug prescription costs (healthcare)
Thomas et al. (2022), United Kingdom [50]	adults aged 16 and over	advertising restrictions on high fat, salt and sugar products	School for Public Health Research (SPHR) diabetes prevention model (version 4)	microsimulation	QALYs, new type 2 diabetes cases, cardiovascular disease events	lifetime healthcare costs (healthcare)
Bates et al. (2022), United Kingdom [51]	individuals with, or without diabetes	behavioural weight loss maintenance	SPHR diabetes prevention model	microsimulation	QALYs	healthcare cost (healthcare)
Nilson et al. (2021), Brazil [52]	adults aged 30 to 79 years	voluntary sodium reduction targets	IMPACT(NCD-Brazil)	microsimulation	cardiovascular disease cases and deaths prevented or postponed	formal and informal healthcare costs (healthcare)
Alonso et al. (2021), United Kingdom [53]	adult population	national and international (WHO) population-level salt intake targets	PRIMEtime-CE Model	multistate lifetable	CVD-related cases averted, life years gained, and QALYs gained	healthcare and social care savings (healthcare)
Aminde et al. (2021), Vietnam [54]	Adults aged 25 and over	Population reduction in salt intake to national and WHO targets	proportional multistate lifetable model	multistate lifetable model, Markov	changes in blood pressure, stroke incidence, deaths, HALYs	healthcare costs (healthcare)
Nilson et al. (2020), Brazil [55]	adult population	population-wide reduction of salt consumption to 5 g/day	Preventable Risk Integrated Model (PRIME)	comparative risk assessment	deaths prevented	direct healthcare cost: inpatient care, outpatient care, and medications (healthcare)
Breeze et al. (2020), United Kingdom [56]	HbA1c 6–6.4%, aged 16, no existing diabetes diagnosis	NHS diabetes prevention programme	SPHR diabetes prevention model	microsimulation	QALYs gained	lifetime cost savings of the NHS diabetes prevention programme (healthcare)
Aminde et al. (2020), Cameroon [57]	adult population (30 years and above)	population salt reduction strategies: mass media campaign, school education programme, low sodium salt substitute	proportional multistate lifetable model	multistate lifetable Markov model	disease incidence, mortality, HALYs	healthcare costs (healthcare)
Mytton et al. (2020), United Kingdom [58]	children aged 0–17 years	HFSS television advertising restrictions between 05.30 h and 21.00 h	PRIMEtime multi-state lifetable model	multistate lifetable model	DALYs	healthcare costs, social care costs, employment costs, health-related net monetary benefits (societal)
Blakely et al. (2020), New Zealand [59]	general population	salt tax	proportional multistate lifetable model	multistate lifetable model	HALYs	health expenditure (healthcare)
Laverty et al. (2019), United Kingdom [60]	adult population	Public Health Responsibility Deal	IMPACT(NCD) model	microsimulation	CVD and gastric cancer cases and deaths	healthcare costs and workplace productivity losses (societal)
Collins et al. (2019), United States of America [61]	adults aged 30 to 84 years	achieving FDA reformulation targets for sodium	US IMPACT Food Policy Model	microsimulation	QALYs gained	health care costs, productivity, informal care, and industry and governmental costs of reformulation (societal)
Briggs et al. (2019), United Kingdom [62]	general population	Salt reformulation	PRIMEtime CE model	multistate lifetable model	QALYs	NHS savings, social care savings, intervention costs (societal / economic)
Kypridemos et al. (2018), United Kingdom [63]	adults aged 30 to 84 years	NHS Health Check programme with additional interventions including mandatory salt reformulation of processed foods	IMPACT(NCD) model	microsimulation	disease cases and deaths prevented or postponed, QALYs gained	programme costs (healthcare and societal)
Basu et al. (2018), the Middle East [64]	Palestinian refugees	alternative food parcels, compared to usual food parcels	microsimulation model	microsimulation	type-2 diabetes, and cardiovascular disease morbidity and mortality, DALYs	healthcare expenditures (healthcare and societal)
Pearson-Stuttard et al. (2018), United States of America [65]	adults aged 30 to 84 years	voluntary sodium reduction	US IMPACT Food Policy Model	microsimulation	CVD deaths and cases prevented or postponed, QALYs, life years gained	medical costs and policy costs (healthcare and societal)
Brown et al. (2018), Australia [66]	general population	legislation to restrict high in fat, sugar and salt (HFSS) TV advertising	proportional multi-state lifetable model	multistate lifetable model	HALYs	healthcare cost savings (societal)
Li et al. (2017), China [67]	families (elderlies aged 65+) with 10-year-old children	school-based education program to reduce salt intake	5-state Markov model	Markov model	QALYs, deaths averted	intervention cost, medical costs (healthcare)
Cobiac et al. (2017), Australia [68]	general population	taxing excess salt in processed foods	proportional multistate lifetable model	multistate lifetable model	DALYs averted	healthcare costs (healthcare)
Webb et al. (2017), global: 183 countries [69]	general population	10% reduction in sodium consumption	comparative risk assessment model	comparative risk assessment	DALYs	intervention costs (healthcare)
Wang et al. (2016), China [70]	adult population aged 35–94 years	national dietary salt reduction goals	CVD Policy Model	Markov model	QALYs; CVD incidence and mortality	health care costs, acute treatment costs, and chronic state costs of CVD (healthcare)
Watkins et al. (2016), South Africa [71]	general population	salt reduction policy	Extended Cost-Effectiveness Analysis salt reduction model	Markov model	cases of catastrophic health expenditure averted, cases of poverty averted, cardiovascular disease cases averted	average out-of-pocket cost of CVD care, treatment costs (societal)
Wilson et al. (2016), New Zealand [72]	adults aged 35 years and over	ten interventions to achieve WHO sodium reduction targets: full target achieved via packaged food target, fast food target and reduced discretionary use; Packaged foods target achieved; Fast food and restaurant target achieved; Bread target achieved; Processed meats target achieved; Sauces target achieved; Snack food target achieved; Cheese target achieved; combination package; all bread and bakery target achieved	Markov macro-simulation model	Markov model	incidence, prevalence and case-fatality rates of CHD and stroke, QALYs	health system costs (healthcare)
Nghiem et al. (2016), New Zealand [73]	population aged 35 years and over	Mandatory sodium substitution in processed foods, and limits on sodium in bread	Markov macro-simulation model	Markov model	QALYs	net health system costs (healthcare)
Wilcox et al. (2015), Syria [74]	adults aged 25 to 84 years	health promotion campaign, labelling of salt content on packaged foods, mandatory reformulation, and combinations	IMPACT CHD model in Syria	comparative risk assessment	life years gained	total policy cost minus healthcare savings (healthcare)
Nghiem et al. (2015), New Zealand [75]	population aged 35 years and over	Counselling, Endorsement label programme, mandatory sodium reduction and reformulation, Mass media campaign, salt tax, Sinking Lid	Markov macro-simulation model	Markov model	QALY, cardiovascular disease mortality rate	health system cost (healthcare)
Mason et al. (2014), Tunisia, Syria, Palestine, Turkey [76]	general population	health promotion campaign, labelling of food packaging, mandatory salt reduction of processed foods (reformulation)	IMPACT CHD model	comparative risk assessment	life years gained, deaths prevented or postponed	cost saved in private sector, public sector, health care (societal)
Collins et al. (2014), United Kingdom [77]	adults aged 25 years and over	Change4Life, Labelling, Mandatory and voluntary reformulation	IMPACT CHD model	comparative risk assessment	life years gained	health care costs, policy and monitoring costs (healthcare)
Ortegon et al. (2012), sub-Saharan Africa and South East Asia [78]	general population	Voluntary or regulatory reduction in dietary salt intake (amongst 123 single or combined prevention and treatment strategies for CVD, diabetes and smoking)	WHO-CHOICE model, including PopMod	comparative risk assessment, multistate lifetable model	DALYs averted	patient and programme costs (healthcare)
Ferrante et al. (2012), Argentina [79]	adult population over the age of 35 to 84	reducing salt content in food by 5–25%	Coronary Heart Disease Policy Model	Markov model	QALY gains	cost of intervention and heart disease (healthcare)
Cobiac et al. (2012), Australia [80]	Australian men and women, aged 35 to 84 years, who have never experienced a CVD event (angina, myocardial infarction, or stroke)	One salt intervention amongst 9 targeting CVD —mandatory reduction of salt in manufacture of breads, margarines and cereals.	discrete time Markov model	Markov model	health gain (DALYs)	intervention costs (healthcare)
Dodhia et al. (2012), United Kingdom [81]	population aged 16 and over	salt reduction in the population, dietary approaches to stop hypertension	Excel spreadsheet simulation model	Markov model	DALYs, YLLs, YLDs, deaths and avoidable deaths	intervention, medical and pharmacological costs (healthcare)
Barton et al. (2011), United Kingdom [82]	Entire population	legislation to reduce salt intake and ban industrial fats	Spreadsheet model	comparative risk assessment	Cardiovascular events avoided, QALYs gained	savings in healthcare costs (healthcare)
Ha et al. (2011), Vietnam [83]	general population	Health education through mass media education to reduce salt intake, and voluntary reduction in salt content of processed foods	WHO-CHOICE, including PopMod	comparative risk assessment, multistate lifetable	DALYs averted	programme and patient-related costs (societal)
Martikainen et al. (2011), Finland [84]	Population aged 30–74 years	reduced daily salt intake and replacement of saturated fat with polyunsaturated fat	state transition Markov cohort model	Markov model	QALYs	direct cost of prevention, morbidity, rehabilitation and production losses due to non-fatal CVD events (societal)
Rubinstein et al. (2010), Argentina [85]	Argentinian population over 35 years old	Voluntary salt reduction in breads	population-level comparative risk assessment	comparative risk assessment	DALYs	intervention costs (healthcare)
Smith-Spangler et al. (2010), United States of America [86]	US adults aged 40–85 years	sodium tax and voluntary sodium reduction in processed foods	computer-simulated, state-transition Markov cohort model	Markov model	QALYs, life years gained, CVD events averted	total savings in medical costs, incremental costs (societal)
Bibbins-Domingo et al. (2010), United States of America [87]	general population	national effort to reduce salt consumption by 3 g per day	Coronary Heart Disease (CHD) Policy Model	Markov model	incidence of CHD, stroke, myocardial infarctions, and deaths from any related cause, QALYs	healthcare savings (healthcare)
Cobiac et al. (2010), Australia [88]	Population aged 30–100 years	dietary advice for everyone or high-risk individuals; incentivised (voluntary) and mandatory moderate salt limits in processed foods	proportional multistate lifetable model	multistate lifetable model	DALYs averted	healthcare and intervention costs (healthcare)
Palar et al. (2009), United States of America [89]	adults aged 18 or older	reduction of population-level dietary sodium consumption	cross-sectional simulation model	comparative risk assessment	QALYs, hypertension prevalence	direct healthcare cost savings (healthcare)
Dall et al. (2009), United States of America [90]	adult population	population-wide sodium intake reduction	Nutrition Impact Model	comparative risk assessment	cases of hypertension	medical cost savings (healthcare)
Akkazieva et al. (2009), Kyrgyzstan [91]	general population	Health education through mass media to reduce daily salt intake	WHO-CHOICE model	comparative risk assessment	DALYs averted	Intervention cost (healthcare)
Rubinstein et al. (2009), Argentina [92]	Population of Buenos Aires	health education through mass media to reduce salt consumption, and voluntary reduction of salt in bread	WHO-CHOICE methodology, and a standard multi-state modelling tool, PopMod	comparative risk assessment, multistate lifetable model	DALYs saved	hospital admission costs, intervention cost, and yearly treatment cost, (government/public sector)
Asaria et al. (2007), 23 countries, including low-income and middle-income countries (Russia, Ukraine, Poland, Turkey, Burma, Indonesia, Egypt, Vietnam, India, South Africa, China, Philippines, Iran, Argentina, Brazil, Bangladesh, Pakistan, Thailand, Colombia, Nigeria, Mexico, DR Congo, Ethiopia) [93]		voluntary reduction in salt content of processed foods, and mass media campaign	WHO Comparative Risk Assessment	comparative risk assessment	deaths averted, cardiovascular mortality	healthcare and intervention costs (healthcare)
Murray et al. (2003), South East Asia, Latin America and Europe [94]	high cardiovascular risk individuals	Salt reduction through voluntary agreements with industry and population-wide reduction in salt intake legislation	PopMod (WHO-CHOICE)	multistate lifetable model	cost per DALYs averted	programme and patient-level costs (healthcare)
Selmer et al. (2000), Norway [95]	1995 Norwegian population aged 40 and over	Health promotion; reformulation, labelling; taxes on salty food/subsidies of products with less salt	dynamic simulation model	Markov model	life years gained	avoided healthcare costs, increased productivity, avoided time loss, cost of health care in extended life, intervention cost, welfare losses (societal)
Joint alcohol and salt policy models						
Bertram et al. (2021), Eastern sub-Saharan Africa and South East Asia [96]	general population	77 interventions for prevention and control of NCDs and MNS (mental, neurological and substance use disorders) including taxation, voluntary and legislative actions to reduce sodium intake	WHO CHOICE	comparative risk assessment, multistate lifetable	Healthy Life Years	patient level delivery costs, programmatic costs and health system costs (healthcare)
Cheatley et al. (2021), 36 countries (South Africa, Japan, Australia, Austria, Belgium, Bulgaria, Croatia, Cyprus, Czech Republic, Denmark, Estonia, Finland, France, Germany, Greece, Hungary, Ireland, Iceland, Italy, Lithuania, Latvia, Malta, Netherlands, Norway, Poland, Portugal, Romania, Russian Federation, Slovak Republic, Slovenia, Spain, Sweden, Switzerland, United Kingdom, Canada, Mexico) [97]	general population	food menu labelling, mass media campaigns, alcohol tax, minimum unit pricing	OECD Strategic Public Health Planning for NCDs (SPHeP-NCDs) model	microsimulation	cancer-related health outcomes (incidence and mortality), DALYs	healthcare costs (healthcare)
Salomon et al. (2012), Mexico [98]	general population	voluntary industry salt reduction and legislation to reduce salt in processed foods; alcohol taxation, reduced access, comprehensive advertising ban, brief advice in primary healthcare	multistate population model PopMod	multistate lifetable model	DALYs	patient, programme and training costs (societal)

### Study characteristics

**Fig. 2:**
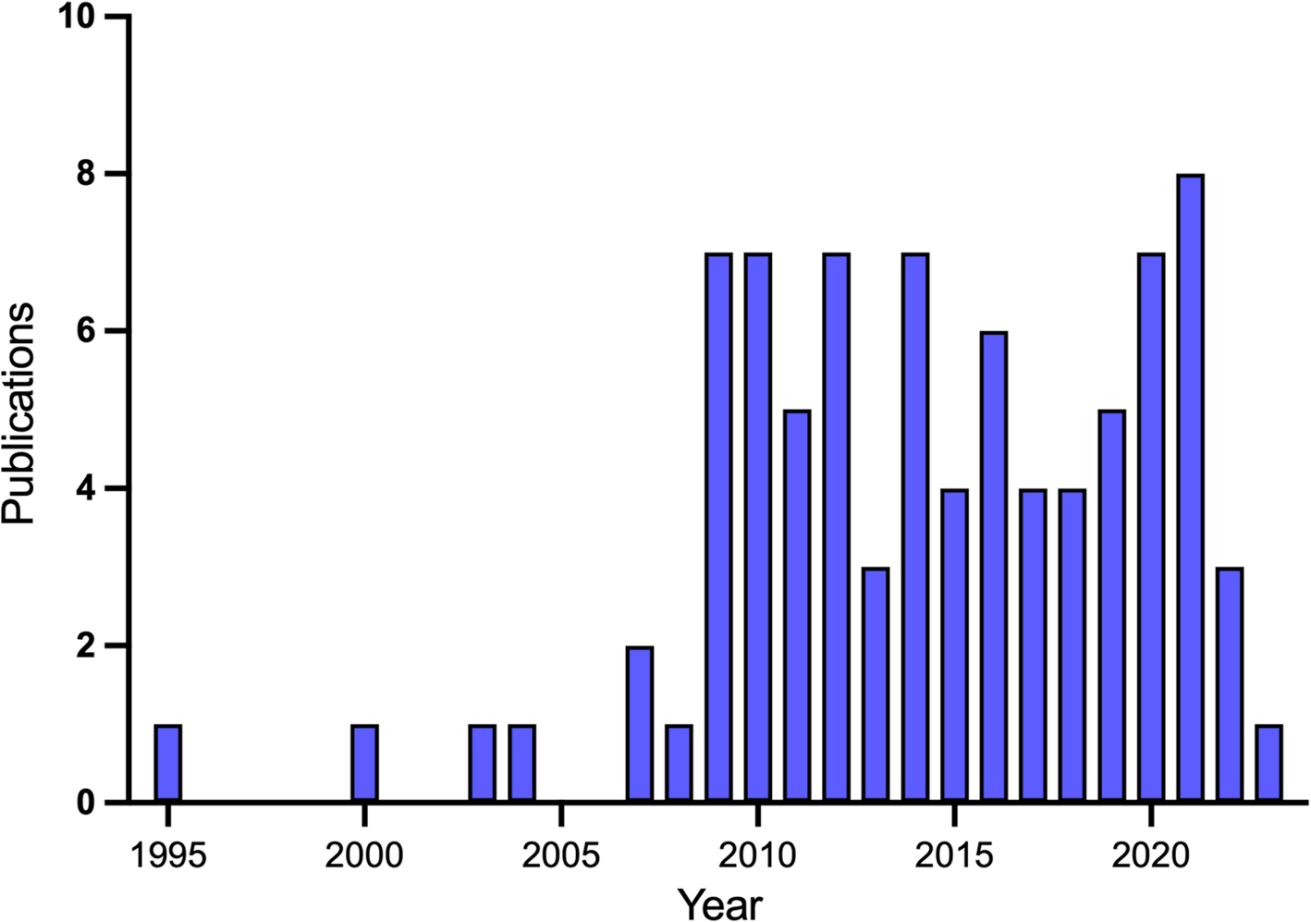
Publication years of included modelling studies

**Fig. 3:**
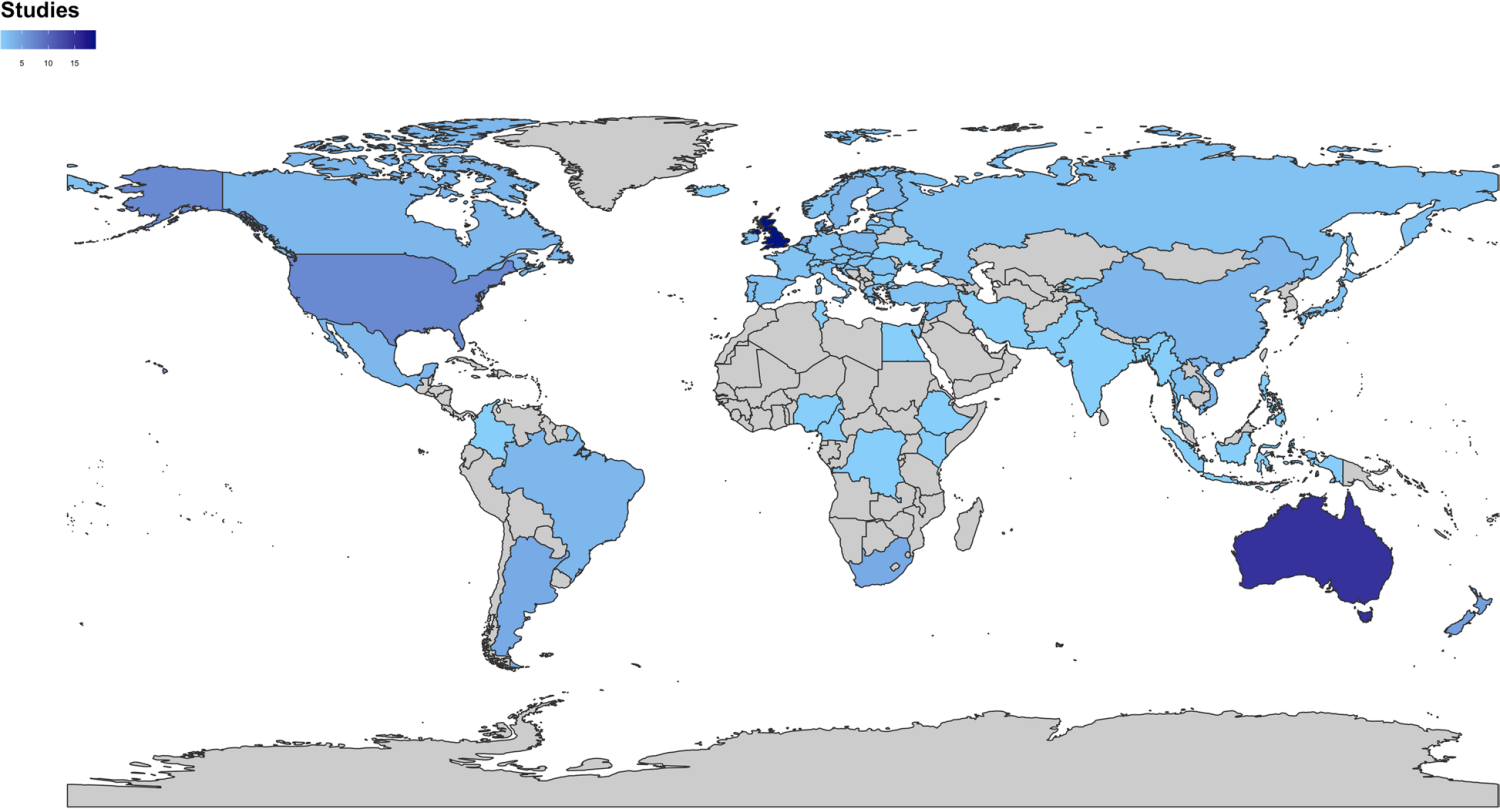
Map showing number of alcohol and salt modelling studies globally

The oldest published study included in the review was published in 1995 [47]. Out of the 82 included studies, only 9% were published between 1995 and 2008. There was an increase in the number of health economic modelling studies published in subsequent years, which has been sustained (Fig. 2). Policies were evaluated in a total of 64 countries globally (Fig. 3), and 66% of all included studies were conducted in high-income countries. Some of these studies conducted evaluations across multiple countries [99] or over specified epidemiological regions [46].

Policy interventions aimed at reducing dietary salt intake, alcohol consumption, or both were most frequently evaluated in the UK and Australia, with 23% and 18% of the studies focusing on these populations, respectively. Among all the included studies, countries in the European region were the most represented, while countries in Sub-Saharan Africa, the Eastern Mediterranean and Southeast Asia were the least represented (Fig. 3). Health economic modelling studies in low- or middle-income countries (LMICs) accounted for only 10% of the included studies.

### Modelled population, interventions and risk factors

The modelled population in the included studies were mostly adults, generally ranging in age from 25 to 85 years. Several alcohol policy models specifically targeted at-risk groups, such as risky drinkers or individuals at high cardiovascular risk, within their evaluation [36, 41, 45]. Two alcohol modelling studies [24, 26] modelled HIV-infected persons and other at-risk populations, although there were no added considerations regarding the intervention effect on the target population. Only one study modelled a population of children aged 0–17 years, assessing the impact of dietary policies on their health over the course of their lives [58].

Most of the modelling studies evaluated at least one policy intervention to reduce salt intake or improve diet (58%), while 38% of the models were specifically developed to evaluate alcohol consumption policies. Only 4% of the included modelling studies evaluated both alcohol and salt-related policies.

**Fig. 4:**
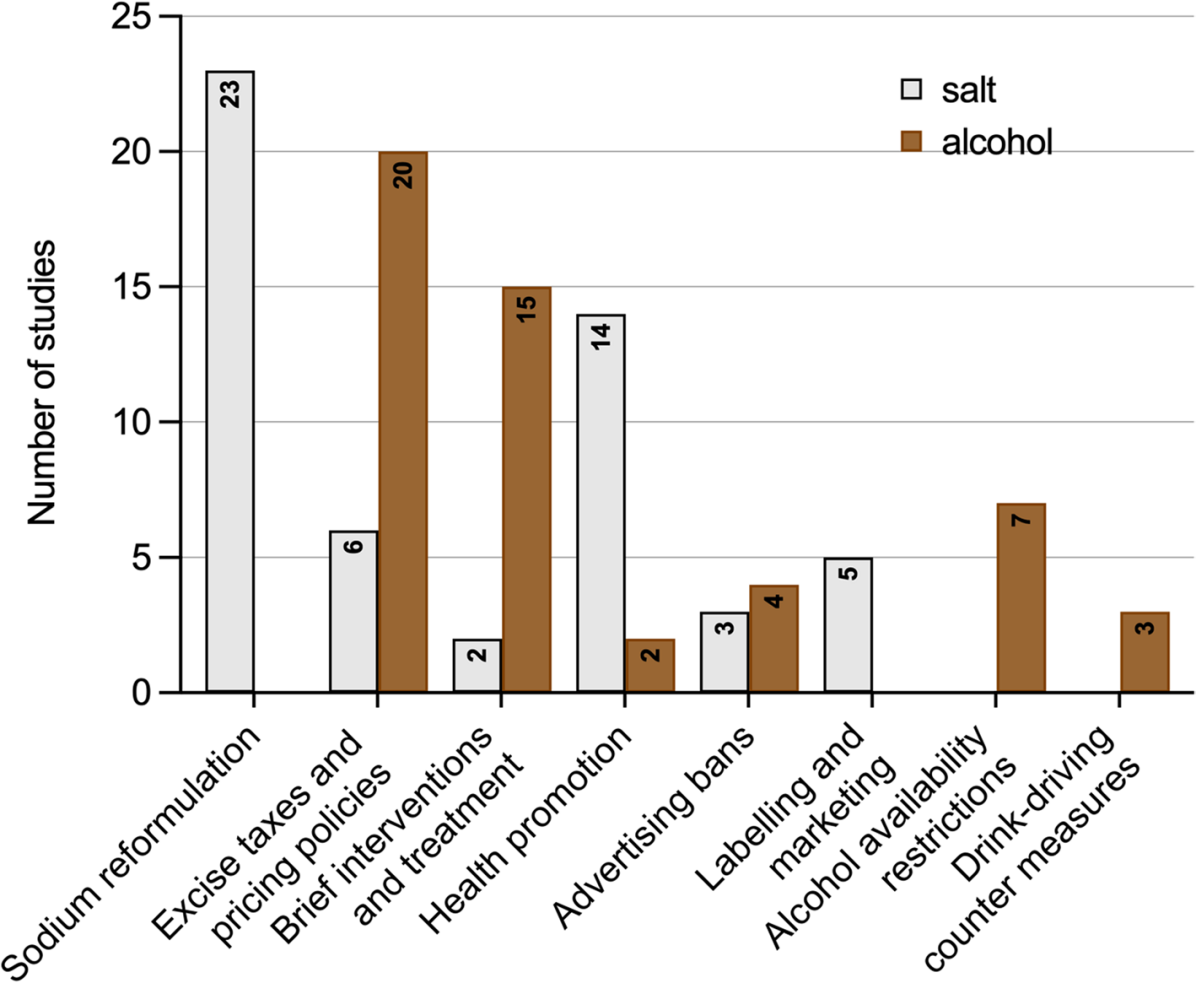
Number of studies evaluating eight categories of salt and alcohol policies examined in the review

The evaluated salt policies included sodium reformulations, sodium taxes, and nutrient labelling (Fig. 4). Sodium reformulation was the most common intervention evaluated (*n* = 23). The effect of these salt-reduction interventions is usually linked to health outcomes through systolic blood pressure and other risk reductions. For example, Wilson et al. [72] determined the risk of coronary heart disease (CHD) and stroke events based on the reduction in systolic blood pressure caused by the evaluated health intervention. Rubinstein et al. [92] also modelled the impact of health promotion campaigns through mass media on reducing salt consumption, focusing on both blood pressure and total cholesterol levels as intermediate risk factors for adverse health outcomes.

On the other hand, alcohol policies such as taxation and minimum unit pricing policies and primary care interventions were the most common interventions evaluated among the alcohol policy modelling studies (Fig. 4). Alcohol consumption frequency was the most common risk factor modelled in these studies, but there was no modelled disease risk pathway through systolic blood pressure from alcohol to health outcomes. The effect of alcohol consumption on the risk of CVD, stroke events, and other alcohol-attributable diseases and complications was modelled directly. These intervention effect sizes are normally derived from observational studies or meta-analyses and are implemented on the target population over a predetermined time horizon.

The review identified only three (4%) studies that modelled both salt and alcohol as independent risk factors for disease in their economic evaluation [96–98]. These studies individually constructed a health economic decision model to evaluate multiple interventions to combat NCDs, including salt-reduction and alcohol taxation policies. Policies that target both salt and alcohol consumption and other risk factors include taxation policies and health promotion interventions. Other risk factors for NCDs that were included in these models included smoking and cholesterol concentration.

Multiple risk factors were therefore incorporated in the health economic models included in this review, including total blood cholesterol levels, body mass index (BMI), type 2 diabetes status, overweight/obese status, drinking history, hypertensive status, and history of other cardiovascular diseases. These risk factors were either assumed to be associated with salt or alcohol consumption or independent of them in relation to the modelled outcomes and diseases.

### Model outcome measures and diseases

Of the 82 included studies, 59 (72%) assumed a healthcare system perspective in the costing approach used in their economic evaluation, while 23 (28%) evaluated from the societal perspective. The healthcare system perspective was defined as a costing perspective amalgamating the healthcare system, government, public sector, and financial cost perspectives. The majority of these studies included direct healthcare, intervention/programme implementation, and informal care costs in their economic analyses. The additional cost components considered from the societal perspective include other economic costs such as productivity gains or losses, health-related net monetary benefits, and avoided time loss. These costs were obtained from publications or estimated by local experts.

Reported health outcomes from economic evaluations include health-adjusted life-year metrics such as quality-adjusted life years (QALYs) gained and disability-adjusted life years (DALYs) averted. Studies also reported epidemiological estimates such as deaths prevented or postponed, disease cases averted, incidence and morbidity (Table 1).

**Fig. 5:**
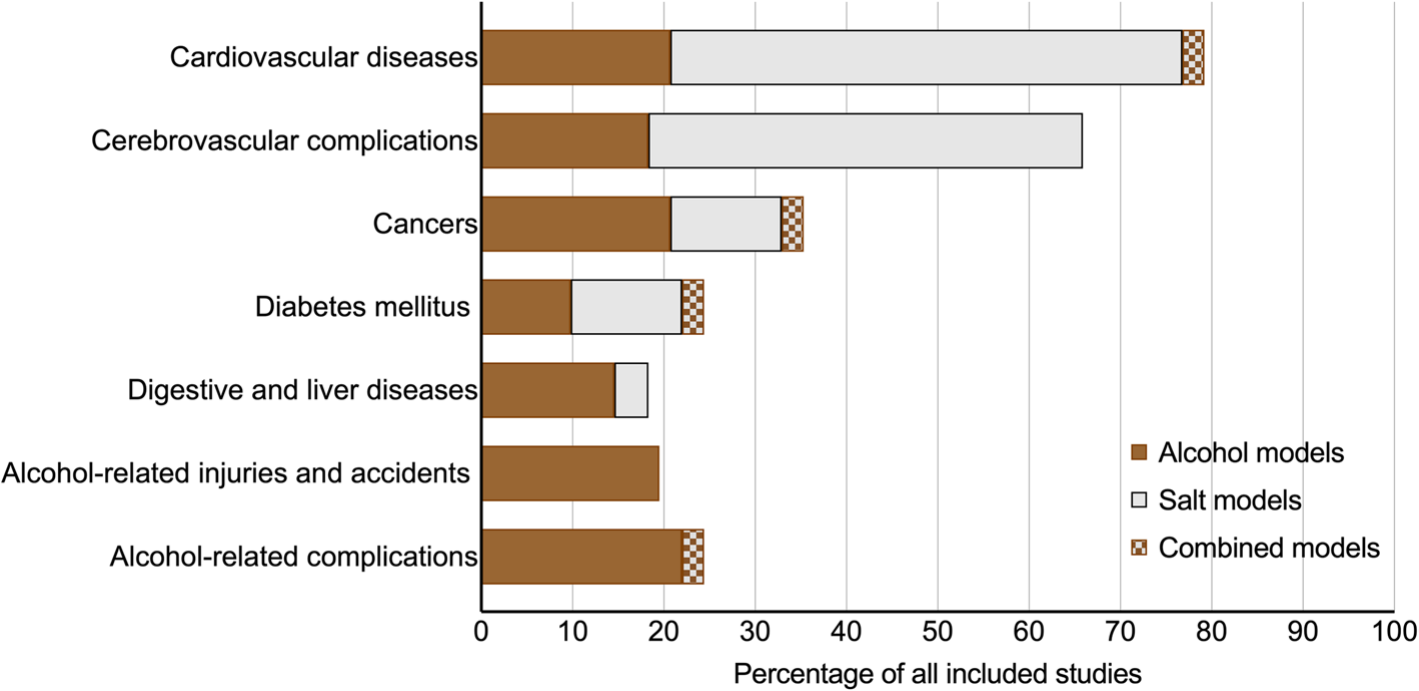
Modelled disease outcomes in included studies

The studies differed in the range of complications and comorbidities considered as outcomes. The range of diseases modelled across the included studies covered the main diet-related cardiometabolic outcomes, cancers, and alcohol-attributable harm (Table 2). The most common diseases reported were cardiovascular diseases (79%), cerebrovascular complications (66%), and cancers (35%), as well as various complications and harms attributable to alcohol (44%) (Fig. 5).

**Table 2:** Adverse health outcomes included in modelling studies

Study	Cardiovascular diseases	Cerebrovascular diseases	Diabetes	Cancers	Digestive and liver diseases	Alcohol-related injuries/accidents	Alcohol-related complications
Gibbs et al. [18]				✓	✓	✓	
Robinson et al. [20]	✓	✓	✓	✓			
Cobiac et al. [22]						✓	
Jiang et al. [21]							✓
Galarraga et al. [24]							
Angus et al. [23]						✓	✓
Zur et al. [25]	✓	✓	✓	✓	✓	✓	
Kessler et al. [26]							
ACE-Prevention model [29, 30]	✓	✓		✓	✓		✓
Doran et al. [33]						✓	✓
Ditsuwan et al. [31]						✓	
Magnus et al. [35]							✓
Popova et al. [34]						✓	✓
Cadilhac et al. [37]	✓	✓		✓			
Navarro et al. [36]							✓
Byrnes et al. [40]	✓	✓		✓	✓		✓
Barbosa et al. [39]							✓
Sheffield Alcohol Policy Model [17, 19, 27, 28, 32, 38]	✓	✓	✓	✓	✓	✓	✓
Cobiac et al. [42]	✓	✓		✓	✓	✓	✓
RIVM Chronic Disease model [41, 43]	✓	✓		✓			
Lai et al. [44]	✓			✓			
Mortimer et al. [45]							✓
Chisholm et al. [46]	✓				✓		
Downs et al. [47]					✓		
Cheatley et al. [97]				✓			
Bertram et al. [96]	✓		✓				✓
Salomon et al. [98]	✓		✓	✓			✓
Aminde et al. [48]					✓		
Ikeda et al. [49]	✓	✓					
Aminde et al. [54]		✓					
Alonso et al. [53]	✓	✓					
Nilson et al. [52]	✓	✓					
Blakely et al. [59]	✓	✓	✓	✓			
Mytton et al. [58]	✓	✓	✓	✓			
Aminde et al. [57]	✓	✓					
SPHR Diabetes prevention model [50, 51, 56]	✓	✓	✓	✓			
Nilson et al. [55]	✓	✓					
Briggs et al. [62]	✓	✓	✓	✓	✓		
Laverty et al. [60]	✓			✓			
Brown et al. [66]	✓	✓	✓	✓			
US IMPACT Food Policy model [61, 65]	✓	✓					
Basu et al. [64]	✓		✓				
Kypridemos et al. [63]	✓	✓	✓				
Cobiac et al. [68]	✓	✓		✓			
Li et al. [67]	✓	✓					
Nghiem et al. [73]	✓	✓					
Wilson et al. [72]	✓	✓					
Watkins et al. [71]	✓	✓					
Wang et al. [70]	✓	✓					
Webb et al. [69]	✓						
Nghiem et al. [75]	✓	✓					
IMPACT CHD model [74, 76, 77]	✓						
Dodhia et al. [81]	✓	✓					
Cobiac et al. [80]	✓	✓					
Ortegon et al. [78]	✓	✓	✓				
Martikainen et al. [84]	✓	✓					
Ha et al. [83]	✓	✓					
Barton et al. [82]	✓	✓					
Cobiac et al. [88]	✓	✓					
Coronary Heart Disease policy model [79, 87]	✓	✓					
Smith-Spangler et al. [86]	✓	✓					
Rubinstein et al. [85]	✓	✓					
Rubinstein et al. [92]	✓	✓					
Akkazieva et al. [91]	✓	✓					
Dall et al. [90]	✓	✓		✓	✓		
Palar et al. [89]	✓	✓					
Asaria et al. [93]	✓						
Murray et al. [94]	✓						
Selmer et al. [95]	✓	✓					

RIVM (the Dutch National Institute for Public Health and the Environment), SPHR (School for Public Health Research), ACE-Prevention (Assessing Cost-Effectiveness in Prevention) model

Among the diseases included in both the salt and alcohol policy models, CHD was the most common modelled cardiovascular disease. The IMPACT CHD model evaluated salt-reduction policies in the UK and Syria populations [74, 76, 77]. The expected change in population salt intake from the interventions was translated into a change in mean blood pressure in the modelled populations. This policy effect was subsequently used to estimate the hypertension prevalence and coronary heart disease deaths prevented or postponed.

Hypertension was modelled explicitly as a health outcome in some of the included salt models; hence, the costs of medication and treatments were included [50, 61, 63, 64, 71]. Hypertension, or systolic blood pressure, was an intermediate risk factor for CVD in 40 (83%) of the salt modelling studies but not in the alcohol policy models. Aminde et al. [54] reported changes in blood pressure after a population reduction in salt intake to WHO targets using a proportional multistate lifetable model. The model then estimates the impact of blood pressure changes on stroke incidence and mortality. This cerebrovascular event was therefore modelled downstream as a complication of high blood pressure and was not directly associated with excessive salt intake.

Other non-hypertensive complications included as explicit health outcomes in the health economic models include diabetes, liver cirrhosis, chronic kidney disease, and various cancers. Cancers which were modelled with salt as a risk factor included breast cancer (in women), gastric, kidney, liver, colorectal, kidney and bowel cancer. These cancers, as well as oesophageal, laryngeal, rectal and oropharyngeal cancer, were also attributable to alcohol consumption in some alcohol policy models. In addition, Brennan et al. [17] reported hospitalisations, crimes and alcohol-attributable deaths and inequalities that result from implementing an alcohol pricing policy, while Gibbs et al. [18] presented changes in alcohol consumption and expenditures when implementing such policies. These health economic outcomes were estimates of the burden of alcohol-related complications and injuries such as road traffic injuries, falls, fires, suffocation, and self-harm.

### Modelling methodology and structures

**Fig. 6:**
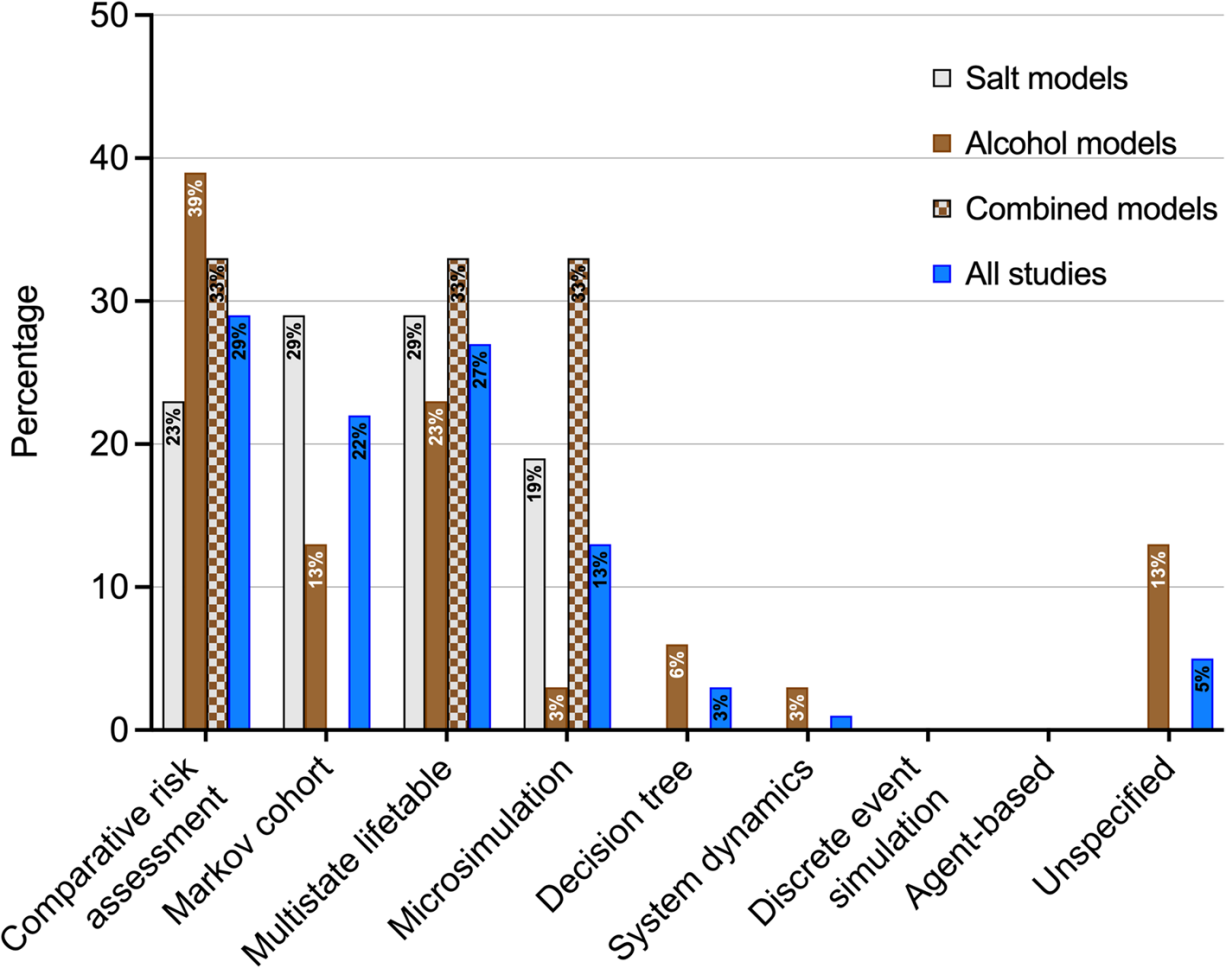
Percentage abundance of structures used in identified models

The models varied widely, from individual-level and cohort state-transition models, to attributable fraction models using a comparative risk assessment method, though none of the studies explored any interaction between individuals. The studies utilised the following epidemiological modelling methods: comparative risk assessments (29%), multistate lifetables (27%), Markov cohort (22%), microsimulation (13%), decision trees (3%), system dynamics models (1%), and other unspecified models (5%) (Fig. 6). There were no agent-based or discrete event simulation modelling studies included in this review. Some of these studies were based on established models that have been applied in different populations to evaluate different policies, with minimal changes in the model structure.

PopMod is one of the first published multistate dynamic lifetable modelling tools that has facilitated disease modelling and cost-effectiveness analysis for priority setting in diverse settings [100]. Murray et al. [94] used this tool to simulate the evolution of a stable population for the cost-effectiveness analysis of interventions to reduce cholesterol and systolic blood pressure in different areas of the world. The structure of this analysis was based on a system of ordinary differential equations that models the temporal evolution of a population subjected to two disease conditions.

Salomon et al. [98] also used the PopMod tool to estimate the cost-effectiveness of various interventions, including salt-reduction and alcohol interventions, to reduce the burden of non-communicable diseases in Mexico. This finding demonstrated the transferability of the model, which not only models interacting disease conditions but also incorporates both alcohol use and salt intake via systolic blood pressure as risk factors for disease. This modelling tool is typically used in the WHO-CHOICE programme [101, 102]. Moreover, comparative risk assessment (CRA) models have been used to estimate the cost-effectiveness of salt and alcohol interventions for combating NCDs in diverse settings in Southeast Asia, Eastern Sub-Saharan Africa, and a range of low- or middle-income countries (LMICs), making the results more comparable [93, 96].

The Dutch National Institute of Public Health and Environmental Protection (RIVM) Chronic Disease model was used to simulate the change in prevalence rates of diseases causally related to alcohol consumption caused by an intervention [41, 43]. The relative risks for diseases related to alcohol consumption were derived from a meta-analysis with estimates for different alcohol consumption categories. The Assessing Cost-Effectiveness (ACE) in Prevention model, developed in Australia, enables a comprehensive cost-effectiveness analysis of preventative intervention options [29, 33]. Over the years, both the ACE-prevention and RIVM models have undergone adaptations to incorporate updated inputs and accommodate changes to their structure [103].

The proportional multistate lifetable Markov model is a dynamic epidemiological model well suited for comparing multiple countries and providing valuable insights for the prioritisation of preventive interventions at the national, regional, and global levels [104]. It has been used to model preventive interventions with the aim of reducing both salt- and alcohol-attributable disease burden [42, 68, 88]. In this model, disease progression is estimated in Markov health states and linked to the multiple cohort lifetable.

This multistate lifetable approach was adopted in the UK for the PRIMEtime Cost-Effectiveness model, which estimates diet changes on morbidity and mortality via blood pressure, cholesterol and body weight [105]. This model facilitated the evaluation of the 2003 to 2018 population salt reduction program implemented in England, with the model projecting its impact by 2050 [53]. The downstream effect of salt intake on systolic blood pressure was modelled, which then estimated the risk of CVD burden and the healthcare and social care utilisation needed.

The IMPACT CHD model, developed in the UK, uses a population-attributable risk fraction approach to estimate the cost effectiveness of salt reduction policies for reducing coronary heart disease in England, Syria, and other Eastern Mediterranean countries [74, 76, 77]. This CRA methodology is commonly applied for alcohol models such as the Sheffield Alcohol Policy Model [106], which also incorporates lifetables in its structure to estimate the effect of alcohol pricing policies such as the minimum unit pricing policy [28, 38]. This model applies the potential impact fraction framework to estimate the impact of changes in alcohol consumption on various alcohol-related harm.

Microsimulation models are becoming increasingly common in the health economics field since they address the limitation of cohort models in capturing the variation in individual characteristics [107]. The School for Public Health Research diabetes prevention model uses this methodology in the evaluation of dietary, lifestyle, and diabetes interventions [50, 51, 56]. The modelled effect of restricting advertising for foods high in fat, salt and sugar was on caloric intake and BMI, so the impact of salt intake on blood pressure was not considered [50]. However, the individual patient model structure allowed heterogeneity in the estimation of HbA1c, systolic blood pressure, cholesterol and BMI as risk factors for disease.

The OECD’s Strategic Public Health Planning Model for Non-communicable Diseases also uses a microsimulation approach to estimate the economic impact of primary prevention policies, such as food menu labelling and alcohol taxes [97]. This microsimulation model addresses all major threats related to non-communicable diseases, such as diabetes, cancer, and cardiovascular diseases, as well as injuries and mental health issues. Additionally, it considers modifiable risk factors such as harmful alcohol consumption, unhealthy diet, physical inactivity, and tobacco use. The country-level risk factor profiles and disease epidemiology data are input into an annual microsimulation to generate outputs on health outcomes, healthcare expenditures, and labour force expenditures.

## Discussion

This study aimed to determine the scope of the published literature and review existing evidence on the modelling methods and structures used in health economic models evaluating salt- and/or alcohol-reduction public health policies. These findings indicate growing interest in using health economic models to evaluate the health and economic impacts of interventions targeting salt or alcohol consumption. The increase in studies since the 2010s is likely linked to the development of key health economic models, such as the ACE-prevention and Sheffield Alcohol Policy models, which have either been adapted or inspired similar models for evaluating health policies. The study also highlights the increasing role of health economic models in evidence-based policymaking, especially in high-income countries.

This review gains value from encompassing studies conducted across low-, middle-, and high-income countries. It highlights the lack of health economic modelling studies in LMICs, where the burden of non-communicable diseases, driven in part by alcohol and salt consumption, is increasing [1]. As a result, the use of health economic modelling tools and evidence to support health priority setting in these countries may be limited. Dotsch-Klerk et al. [8] suggested that the limited availability of data, such as epidemiological data and cost data, in lower income countries may explain the limited number of modelling studies from those countries. This is of concern because generating the cost-effectiveness evidence of primary prevention strategies, which is critical in settings with limited financial resources, could be crucial for reducing the burden of NCDs.

The World Health Organisation Choosing Interventions that are Cost-Effective (WHO-CHOICE) programme has been instrumental in such cost-effectiveness analyses of health interventions globally [108]. The use of standardised methods across disease areas is a major added value of the CHOICE approach, as it allows for fair comparisons between and across health programmes. The WHO-CHOICE has developed a software tool, the OneHealth tool (https://www.who.int/tools/onehealth), to help analysts and decision-makers conduct country-based and regional cost-effectiveness analyses of various health interventions for strategic planning and costing. This tool will enable the use of health economic modelling in LMICs to assess the cost-effectiveness of different health strategies for priority setting and resource allocation.

There is also potential for developing joint dietary salt and alcohol policy models that evaluate public health policies targeting both risk factors, exploring their association or integrated effects on health and economic outcomes. Such analyses would require input data (or assumptions) on the joint effects of salt and alcohol consumption, potentially derived from observational studies, clinical models or RCTs. These models could support policymaking aimed at reducing disease burden and assist in priority setting, particularly in resource-constrained settings. For example, health-related food taxes could be evaluated across different dietary behaviour targets, helping decision-makers compare cost-effectiveness across various policies.

The strength of this review lies in its potential to inform the development of public health economic models for evaluating alcohol and salt reduction strategies. By identifying and examining common modelling pathways and approaches, it provides a foundation for adapting these methods for future models. This review explores the effects of alcohol and dietary salt reduction interventions on health outcomes, both directly and through intermediate risk factors such as systolic blood pressure, summarising the various modelling pathways used. While a detailed review of the epidemiological studies estimating the effects of these intermediate risk factors on health outcomes would be valuable, it was beyond the scope of this review.

Previous systematic reviews of salt or alcohol models have provided an overview and critically appraised economic models that have served their purpose in informing policymakers about the availability of modelling techniques [6]. However, to our knowledge, no studies have compared the differing modelling structures of alcohol and salt models, or explored their risk factor pathways to disease. Furthermore, the systematic approach used for the literature search and data collection ensures rigour.

A limitation of this review is that it did not assess the quality of the identified economic evaluations, nor did it include a critical analysis of the models in regard to the appropriateness of modelling methods in health economic studies, as this was not the intended purpose of the review. The purpose of this scoping review was to provide an overview of the health economic models and the methods used. Therefore, a systemic evaluation of the included studies and a risk of bias assessment were not needed. Another limitation is that only studies published in English were included, which means that methods used by other studies published in other languages might have been missed.

Nonetheless, the review findings can inform economic evaluation methods for analysing the cost-effectiveness of salt and alcohol public health policies. The evidence base generated from health economic models can underpin policymaking on alcohol and salt reduction in the population. Future research should prioritise the development of health economics models that incorporate multiple risk factors of disease and are broadly applicable for evaluating related interventions. Such models would enable cost-effectiveness analyses of policies targeting multiple risk factors, such as alcohol and salt, to support priority setting.

## Conclusions

This scoping review identified modelling studies used to evaluate policies and lifestyle interventions to reduce salt and/or alcohol intake. It offers valuable insights into the diverse public health economic modelling approaches, highlighting their varying complexities and information requirements in estimating the health and economic impacts of these interventions. This review revealed that systolic blood pressure was a key intermediate risk factor in the excessive salt-to-disease modelling pathway for most studies. However, the effects of alcohol consumption on adverse health effects are usually modelled directly using estimates of the relative risk of disease. We identified only a few modelling studies that incorporate both alcohol and salt as risk factors. It is also clear from this review that incorporating multiple risk factors in health economic model evaluations, especially in LMICs where it’s limited, will generate the needed cost-effectiveness evidence for decision makers, to facilitate the implementation of policies to reduce both salt and alcohol consumption.

## Supplementary Information

Supplementary Material 1.

## Data Availability

All data generated or analysed during this study are included in this published article [and its supplementary information file].

## Abbreviations

ACE: Assessing Cost-Effectiveness
BMI: Body mass index
CHD: Coronary heart disease
CRA: Comparative risk assessment
CVD: Cardiovascular disease
DALY: Disability-adjusted life year
DES: Discrete event simulation
LMIC: Low- or middle-income country
NCD: Non-communicable disease
QALY: Quality-adjusted life year
RCT: Randomised controlled trial
RIVM: National Institute for Public Health and the Environment
WHO: World Health Organisation
