# Supplementary material for: Public health economic modelling in evaluations of salt and/or alcohol policies: a systematic scoping review
Source: BMC Public Health. 2025 Jan 8;25:82. doi: 10.1186/s12889-024-21237-7 (PMC11707988; doi:10.1186/s12889-024-21237-7)
Supplement: Supplementary file 1 — Supplementary Material 1. [file 12889_2024_21237_MOESM1_ESM.docx]

**Appendix 1**: Search strategy

MEDLINE search strategy, which was modified for the other electronic databases (EMBASE and EconLit).

| **Literature Search performed: 8^th^ November 2023** | | **Results** |
| --- | --- | --- |
| 1 | salt.mp. | 177473 |
| 2 | Salts/ | 14862 |
| 3 | Sodium/ or exp Sodium, Dietary/ | 123454 |
| 4 | sodium.mp. or Sodium/ or exp Sodium, Dietary/ or exp Sodium Chloride, Dietary/ | 595822 |
| 5 | alcohol.mp. | 357450 |
| 6 | exp Alcohol Drinking/ | 78560 |
| 7 | exp Alcoholism/ or binge drinking.mp. or exp Binge Drinking/ | 86853 |
| 8 | exp Alcoholic Intoxication/ | 13069 |
| 9 | alcohol consumption.mp. | 52752 |
| 10 | economic evaluation.mp. or Cost-Benefit Analysis/ | 99220 |
| 11 | model*.mp. | 4850790 |
| 12 | Computer Simulation/ | 212158 |
| 13 | Models, Theoretical/ | 162611 |
| 14 | Economics, Dental/ or Economics, Hospital/ or Economics/ or Economics, Pharmaceutical/ or Economics, Nursing/ or Economics, Medical/ | 56355 |
| 15 | exp "Costs and Cost Analysis"/ | 267088 |
| 16 | (economic$ or cost or costs or costly or costing or price or prices or pricing or pharmacoeconomic$).ab,ti. | 1071971 |
| 17 | (expenditure$ not energy).ab,ti. | 37671 |
| 18 | value for money.ab,ti. | 2177 |
| 19 | budget$.ab,ti. | 36268 |
| 20 | 14 or 15 or 16 or 17 or 18 or 19 | 1236803 |
| 21 | ((energy or oxygen) adj cost).ab,ti. | 4834 |
| 22 | (metabolic adj cost).ab,ti. | 1742 |
| 23 | ((energy or oxygen) adj expenditure).ab,ti. | 29480 |
| 24 | 21 or 22 or 23 | 34978 |
| 25 | 20 not 24 | 1228715 |
| 26 | letter.pt. | 1234466 |
| 27 | editorial.pt. | 670966 |
| 28 | historical article.pt. | 369446 |
| 29 | 26 or 27 or 28 | 2253869 |
| 30 | 25 not 29 | 1188407 |
| 31 | exp animals/ not humans/ | 5168407 |
| 32 | 30 not 31 | 1111831 |
| 33 | 1 or 2 or 3 or 4 | 729106 |
| 34 | 5 or 6 or 7 or 8 or 9 | 395394 |
| 35 | 11 or 12 or 13 | 4891114 |
| 36 | 10 or 32 | 1121365 |
| 37 | 33 or 34 | 1114278 |
| 38 | 35 and 36 and 37 | 5930 |
| 39 | 10 and 35 and 37 | 478 |
| 40 | 10 and 32 | 89686 |
| 41 | 35 and 37 and 40 | **474** |
